# Anti-Photodamage Effect of *Agaricus blazei* Murill Polysaccharide on UVB-Damaged HaCaT Cells

**DOI:** 10.3390/ijms25094676

**Published:** 2024-04-25

**Authors:** Wenjing Cheng, Feiqian Di, Luyao Li, Chunhong Pu, Changtao Wang, Jiachan Zhang

**Affiliations:** 1 School of Light Industry Science and Engineering, Beijing Technology & Business University, Beijing 100048, China; 2 Beijing Key Lab of Plant Resource Research and Development, Beijing 100048, China; 3 Institute of Cosmetic Regulatory Science, Beijing 100048, China

**Keywords:** *Agaricus blazei* Murill polysaccharide (ABP), structural characterization, UVB-damaged model, anti-photodamage, antioxidation, inflammation

## Abstract

UVB radiation is known to induce photodamage to the skin, disrupt the skin barrier, elicit cutaneous inflammation, and accelerate the aging process. *Agaricus blazei* Murill (ABM) is an edible medicinal and nutritional fungus. One of its constituents, *Agaricus blazei* Murill polysaccharide (ABP), has been reported to exhibit antioxidant, anti-inflammatory, anti-tumor, and immunomodulatory effects, which suggests potential effects that protect against photodamage. In this study, a UVB-induced photodamage HaCaT model was established to investigate the potential reparative effects of ABP and its two constituents (A1 and A2). Firstly, two purified polysaccharides, A1 and A2, were obtained by DEAE-52 cellulose column chromatography, and their physical properties and chemical structures were studied. A1 and A2 exhibited a network-like microstructure, with molecular weights of 1.5 × 10^4^ Da and 6.5 × 10^4^ Da, respectively. The effects of A1 and A2 on cell proliferation, the mitochondrial membrane potential, and inflammatory factors were also explored. The results show that A1 and A2 significantly promoted cell proliferation, enhanced the mitochondrial membrane potential, suppressed the expression of inflammatory factors interleukin-1β (IL-1β), interleukin-8 (IL-8), interleukin-6 (IL-6), and tumor necrosis factor α (TNF-α), and increased the relative content of filaggrin (FLG) and aquaporin-3 (AQP3). The down-regulated JAK-STAT signaling pathway was found to play a role in the response to photodamage. These findings underscore the potential of ABP to ameliorate UVB-induced skin damage.

## 1. Introduction

UVB radiation, with a wavelength ranging from 290 to 320 nm, is a critical component of solar radiation that passes through the atmosphere, ultimately reaching the epidermal layer of the skin. The interaction of UVB radiation with human skin can have a range of harmful effects, encompassing sunburn, tanning, immunosuppression, photoaging, and a heightened risk of skin cancer [1,2,3,4]. The cumulative impact of acute and chronic UV exposure is known to disrupt the structural integrity of the skin, a hallmark manifestation being the process of photoaging [5]. Of significant concern is the generation of reactive oxygen species (ROS), potent molecular agents inflicting oxidative injury on essential skin constituents, including proteins, lipids, and DNA, thus perpetuating the aging phenomenon [6]. 

*Agaricus blazei* Murill polysaccharide (ABP) serves as the main bioactive component intrinsic to *Agaricus blazei* Murill (ABM). The dominant constituent of ABP is glucan, predominantly comprising β-glucan, followed by α-glucan. The noteworthy active constituents are β-D-glucan, β (1,3)-D-glucan, β (1,4)-D-glucan, and β (1,6)-D-glucan [7]. Many effects of ABP have been reported, such as anti-tumor [8], antivirus [9], immune-modulatory [10], and antioxidant [11] attributes. Clinical reports have further proved its therapeutic efficacy in the treatment of cancer, diabetes, and chronic hepatitis [12]. ABP exerts excellent free radical scavenging activities, thereby mitigating oxidative stress [13].

A pivotal facet of ABP’s action lies in its contribution to relieving inflammation. Evidential findings reveal its capability to down-regulate IL-1 levels within rat skin, thus engendering discernible anti-inflammatory responses [14]. Notably, ABP has demonstrated the ability to curtail UV-induced photodamage and enhance cell viability within specific concentration thresholds [15]. Nonetheless, the scientific literature has been limited to some extent in terms of exploring ABP’s potential as an ameliorative agent against UV-induced damage.

The JAK-STAT signaling pathway is associated with growth, survival, development, and differentiation in a variety of cells. It is also crucial to immune function [16]. The dysregulation of this signaling pathway can lead to persistent inflammation and autoimmune diseases. It has been reported that the inhibition of the JAK-STAT signaling pathway is involved in alleviating inflammatory responses [17,18,19,20]. Whether this pathway plays a role in the anti-photodamage effect of ABP needs to be discussed.

In this study, a UVB-induced HaCaT photodamage model was established. Our primary research objectives encompassed the isolation, purification, and structure analysis of polysaccharides A1 and A2, as well as an in-depth exploration of the potential reparative effects wielded by ABP against UVB-induced photodamage. The effects of A1 and A2 on cell proliferation, the mitochondrial membrane potential, and cellular matrix proteins, such as filaggrin (FLG) and aquaporin-3 (AQP3), were discussed. The inflammatory factors IL-1β, IL-8, IL-6, and TNF-α were also measured. Furthermore, the related biomarkers of the JAK-STAT signaling pathway were detected by RT-qPCR, Western blot, and immunofluorescence. We hope this study will provide a theoretical basis for ABP’s application in the treatment of anti-photodamage.

## 2. Results

### 2.1. Effects of ABP on Cell Viability, Inflammatory Cytokine Secretion, and Skin Barrier Function

**Figure 1:**
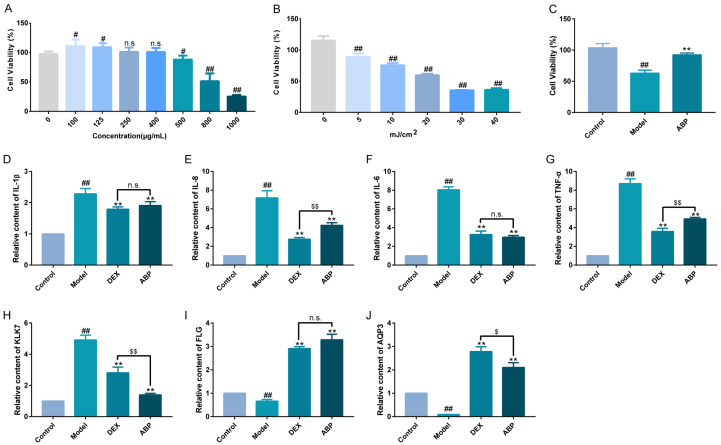
The toxicities of ABP (**A**) and UVB radiation (**B**) and repairing effect (**C**) on HaCaT cells, and the relative contents of IL-1β (**D**), IL-8 (**E**), IL-6 (**F**), TNF-α (**G**), KLK7 (**H**), FLG (**I**), and AQP3 (**J**). DEX at a concentration of 1 μM was used as the positive control. The results are expressed as means ± SD (n = 3). ^#^ *p* < 0.05 and ^##^ *p* < 0.01 compared with the control; ** *p* < 0.01 compared with the model; ^$^ *p* < 0.05 and ^$$^ *p* < 0.01 compared with A1 group; and ^n.s.^ *p* > 0.05.

Figure 1A shows the toxicities of ABP ranging from 0 to 1000 μg/mL in HaCaT cells and suggests that ABP had a good promoting effect on cell proliferation at low concentrations (100~125 μg/mL). In this study, ABP at a concentration of 500 μg/mL was chosen for the following study because the cell viability at that concentration reached up to 88.07 ± 2.76%. Figure 1B shows the viabilities of HaCaT cells treated with different doses of UVB radiation. Exposure to UVB radiation triggered a reduction in cell viability and induced cellular photodamage. Generally, the IC50 parameter was usually selected as the model’s establishing condition [21]. UVB radiation at a dosage of 20 mJ/cm^2^ was used herein to establish the photodamage model. ABP at 500 μg/mL significantly enhanced the UVB-treated cell viability (Figure 1C).

Skin epidermal cells can modulate inflammatory chemokine secretion to maintain balance, involving interleukins and tumor necrosis factors [22]. UVB exposure disrupts this balance by increasing the secretion of these chemokines, leading to skin inflammation and damage [23]. Elevated intracellular interleukin and tumor necrosis factor secretion signifies the initiation of cellular inflammatory responses [24]. Figure 1D–G illustrates the relative contents of pro-inflammatory cytokines IL-1β, IL-8, IL-6, and TNF-α in response to ABP treatment in photodamaged cells. DEX served as a positive control, revealing ABP’s effect on these cytokines [25,26,27]. In this study, UVB irradiation caused the high secretion of pro-inflammatory factors (IL-1β, IL-8, IL-6, and TNF-α), which was consistent with the literature reported [28]. These changes in tendency were also common in other cell types, such as HaCaT cells [29], human epidermal melanocytes [30], and human cord blood-derived mast cells. The DEX and ABP groups exhibited substantially lower content levels compared with the model group.

Functional proteins in the skin cuticle, such as AQP3 and FLG, along with KLK-7, play vital roles in skin barrier integrity [31,32]. UVB-induced AQP3 down-regulation in the basal layer of the epidermis has been demonstrated to have a relationship with the reduction in stratum corneum hydration and glycerol content [33,34]. Post-UVB irradiation, the contents of AQP3 (Figure 1H) and FLG (Figure 1I) in the model group were both decreased, while the content of KLK7 (Figure 1J) was significantly increased. The findings of a previous study have also substantiated this result [35]. Meanwhile, the DEX and ABP treatments induced a marked increase in the AQP3 and FLG contents and a significant decrease in the KLK7 content. These findings highlight UVB’s disruption of the water and protein balance in epidermal cells, resulting in cellular damage and a compromised skin barrier. Some active ingredients, such as trans-zeatin, retinoids, and nicotinamide, have shown a moisturizing function by increasing the AQP3 content [36,37]. Moreover, some herbal extracts, for example, coix seed extract [38], were reported to have the ability to enhance the expression of AQP3. Meanwhile, it is noted that an association between high AQP3 levels and skin tumor formation has been reported [39,40]. Caution seems warranted when discussing the usage of ingredients that could increase epidermal AQP3 expression.

KLK7 is a serine protease encoded by the KLK7 gene located on chromosome 19q13. KLK7 was previously shown to induce inflammation, and increased KLK7 expression in the epidermis of atopic dermatitis patients was observed [41]. In this study, we observed that the changes in KLK7 and IL-1β were consistent in both the model group and the sample group, respectively. One possible mechanism that has been reported is that KLK7 mediates the conversion of pro-IL-1β to active IL-1β [42]. Interestingly, ABP showed significantly better effects than DEX regarding the levels of KLK7 and FLG in this study. This might be because of the structure and physicochemical properties of ABP.

Notably, the results indicate the potential of ABP to mitigate the secretion of inflammatory factors and maintain skin barrier integrity.

### 2.2. Isolation, Purification, and Structural Analysis

**Figure 2:**
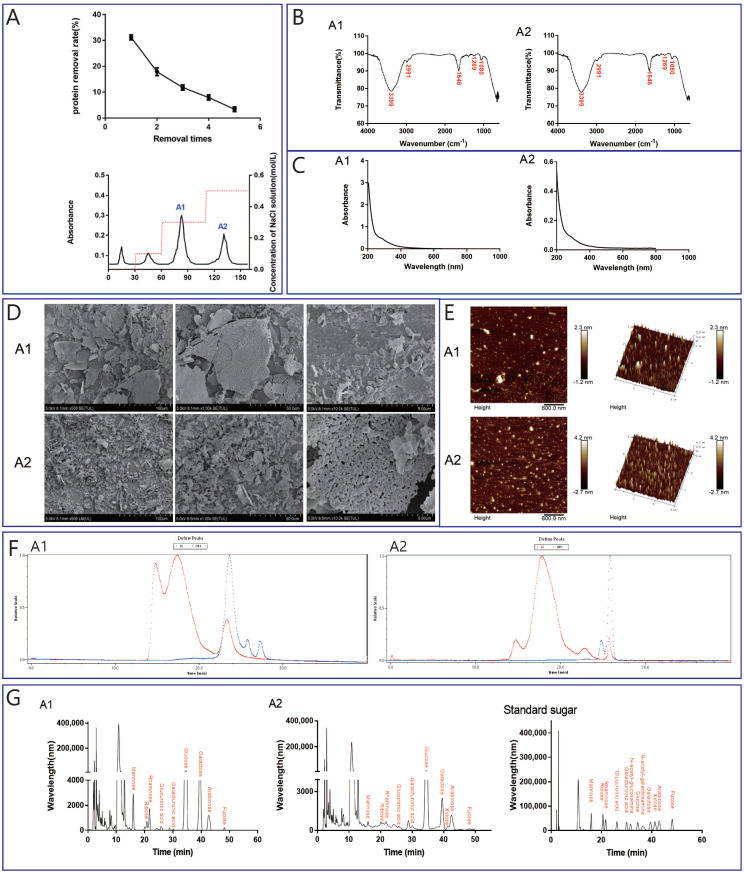
Isolation, purification, and structural characterization of ABP. Isolation and purification (**A**), UV–Vis photometric analysis (**B**), FT-IR-based structural characterization (**C**), SEM (**D**) (5×, 50×, and 100×) and AFM analysis (**E**), GPC-based molecular weight analysis (**F**), and HPLC-based monosaccharide composition analysis (**G**).

The Sevage method was chosen in this study to eliminate the protein from ABP and was replicated five times to ensure a thorough protein removal. As shown in Figure 2A, the results demonstrate a consistent reduction in the protein content in the polysaccharides with each iteration, ultimately yielding a final protein content below 5%. There were two obvious distinct elution peaks we could observe in the elution curve (Figure 2A), designated as A1 and A2. 

Structural analysis of A1 and A2 using UV–visible and FT-IR spectra was employed (Figure 2B,C). The absence of distinct peaks between 200 and 600 nm in the UV spectra of A1 and A2 signified the successful removal of protein and nucleic acid contaminants, indicating the purity of the extracted compounds. Notably, absorption peaks at 3399 cm^−1^ corresponded to the stretching vibration of hydroxyl groups (O-H) [43,44], indicating the presence of these functional groups. The peaks at 2954 cm^−1^ and 2991 cm^−1^ represented the tensile vibrations of C-H bonds [25,45], while the 1646 cm^−1^ peak suggested carbonyl (C=O) stretching vibrations [46]. The peaks at 1420 cm^−1^ indicated the presence of a carboxyl group (COOH) [47], and the absorption peaks at 1261 cm^−1^ and 1269 cm^−1^ indicated C-O-C bonds. Additionally, the presence of an absorption peak at 1080 cm^−1^ raised the possibility of sugar or glycosidic bonds [48], potentially in a pyranose form. The appearance of this absorption peak led us to speculate that A1 and A2 may contain sugar or glycosidic bonds in the form of pyranose. The presence of comparable absorption peaks was also documented in our prior investigation [25,43]. The similarity between the FT-IR spectra of A1 and A2 suggested common major functional groups, such as carboxyl and hydroxyl groups, which contributed to the distinctive physical properties of these components [49]. 

SEM analysis further elucidated the morphologies of A1 and A2. The SEM images obtained at different magnifications (5×, 50×, and 100×) revealed the irregular sheet/network structures of A1 and A2, characterized by smooth and finely textured surfaces. Notably, A1 exhibited larger sheet structures compared with A2, while A2 displayed a more porous microstructure, indicating potential differences in water retention and rheological properties [25,43].

AFM revealed the intermolecular forces and structural changes, providing the surface topographies and ultrastructures [25]. The roughness observed on the surfaces of A1 and A2 suggested their potential biological activities, with A2 exhibiting greater roughness, indicating an enhanced biological potential compared with A1. Three-dimensional (3D) stereographic representations showed that A1 presented dense grain heap-like structures, while A2 displayed pronounced high-pointed and irregular protrusions (Figure 2E). Importantly, the thickness of the polysaccharide chains in A1 and A2, ranging from 0.6 to 2.3 nm and 0.8 to 4.2 nm, respectively, exceeded the dimensions of single-stranded polysaccharide molecules (0.1–1.0 nm). This observation suggested the presence of intertwined intra- and intermolecular forces, including van der Waals forces and hydrogen bonds, contributing to the formation of polymeric structures in A1 and A2 [50]. This indicated that the intra- and intermolecular van der Waals forces and hydrogen bonds in A1 and A2 intertwined to form polymers.

The relative molecular weights of A1 and A2 were determined by gel permeation chromatography (GPC) (Figure 2F). The weight-average molecular weight (Mw) values of A1 and A2 were 1.5 × 10^4^ Da and 6.5 × 10^4^ Da, respectively (Table 1). The peak retention times of A1 and A2 were 21.921–24.953 min and 25.237–26.727 min, respectively. The molecular weights of most polysaccharides in mushrooms are between 10 and 8 × 10^5^ Da, which includes the fractions of ABP in this study. However, some polysaccharides have molecular weights as high as 1 × 10^8^ Da or as low as 4 × 10^3^ Da [51].

Figure 2G and Table 2 show the monosaccharide compositions of A1 and A2. Both A1 and A2 were dominated by glucose, galactose, and arabinose. In addition, A1 contained mannose and rhamnose, and A2 contained low levels of xylose. The top three contents of glucose, galactose, and arabinose in A1 were 61.478%, 21.46%, and 5.832%, respectively. The contents of these three monosaccharides in A2 were 78.373%, 11.108%, and 6.925%, respectively. Glucose is the basic structural unit of many polysaccharides, such as polysaccharides from *Tricholoma magnivelare* [52], *Pleurotus eryngii* [53], *Ganoderma lucidum* [54], etc. The monosaccharide composition of ABP in this study was consistent with this. Apart from glucose, this polysaccharide, which has certain health functions, such as an anti-tumor function, is mainly composed of galactose, mannose, arabinose, rhamnose, and xylose [52]. In this study, the total percentage of these kinds of monosaccharides (galactose, mannose, arabinose, and rhamnose) in A1 was up to 34.287%, which was much higher than that in A2, indicating potential differences between the two in their functions. 

Furthermore, it was reported that mannose helps to regulate the skin’s microecological balance [55], so it is speculated that ABP has skin conditioning activity. It was also reported that fucose frequently plays an important role in bioactivities [56,57]. However, there were low ratios of fucose in both of the two fractions.

### 2.3. Effects of ABP Components A1 and A2 on Cell Viability and Proliferation

**Figure 3:**
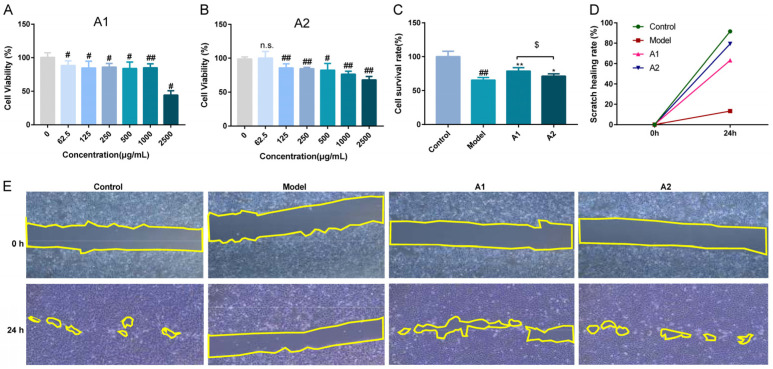
The toxicities of A1 (**A**) and A2 (**B**), and the repairing effects (**C**) of A1 and A2 on the proliferation of HaCaT cells damaged by UVB irradiation (n = 3). (**D**) The cell scratch healing rate of A1 and A2. (**E**) Representative images of effects of A1 and A2 on cell migration (4×), the region delineated by the yellow line exhibited no cellular proliferation. ^#^ *p* < 0.05 and ^##^ *p* < 0.01 compared with the control; * *p* < 0.05 and ** *p* < 0.01 compared with the model; ^$^ *p* < 0.05 compared with A1 group; and ^n.s.^ *p* > 0.05.

To explore the proliferative potentials of A1 and A2 in UVB-damaged cells, the cell viabilities of cells treated with A1 and A2 following UVB exposure were discussed. Our findings indicate that the cell viabilities significantly decreased after UVB damage compared with the blank group. Incubation with A1 and A2 for 24 h significantly increased the cell viabilities in comparison with the model group (Figure 3C). Furthermore, the reparative effect of A1 was more prominent than that of A2.

The process of cell migration is usually a response to extracellular signals, including diffusion factors, signals in adjacent cells, and signals from the extracellular matrix [58]. As far as we know, no report has discussed the potential effects of *Agaricus blazei* on wound healing. 

A cell scratch assay was employed to investigate the effects of A1 and A2 on cell migration. As shown in Figure 3E, the level of cell migration in cells treated with A1 and A2 increased at 24 h under the same conditions compared with the model group. The cell scratch healing rate was measured as the index shown in Figure 3D,E. As shown in Figure 3D, the level of cell migration in cells treated with A1 and A2 increased at 24 h under the same conditions compared with the model group. As shown in Figure 3D, the scratch healing rate at 24 h was highest in the blank group, which was without UVB exposure, followed by the A2 and A1 groups post-UVB treatment. The scratch healing rate of the model group was 11.53% at 24 h, the lowest level in the cell scratch assay. These results indicate that A1 and A2 can increase the level of cell migration, while UVB radiation can reduce the level of cell migration.

### 2.4. Effects of A1 and A2 on Mitochondrial Membrane Potential (ΔΨm)

ΔΨm is an essential parameter for evaluating mitochondrial function, often indicative of apoptotic processes [59]. The differences in the mitochondrial membrane potential observed via JC-1 staining provide valuable information about the cellular health status, with healthy cells demonstrating a higher mitochondrial membrane potential, while unhealthy cells exhibit lower levels [60].

**Figure 4:**
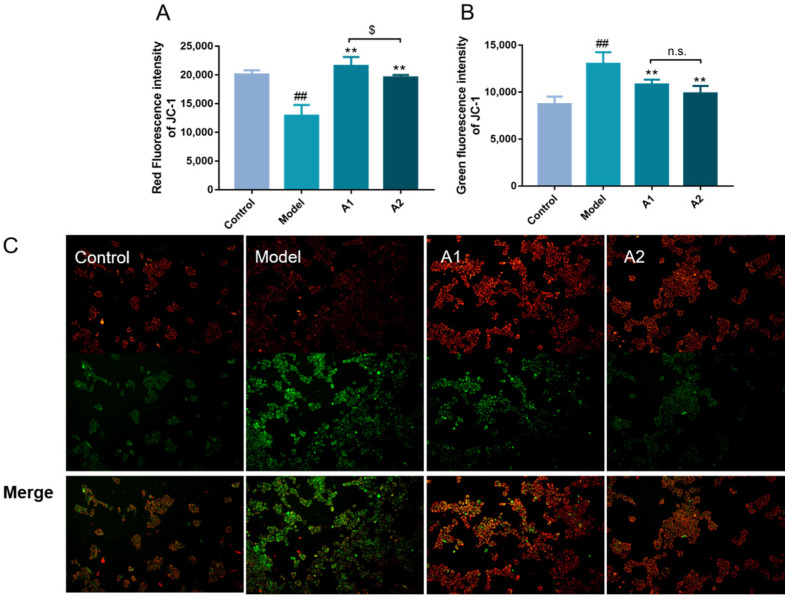
The effects of A1 and A2 on JC-1. Red fluorescence value (**A**), green fluorescence value (**B**), and representative JC-1 fluorescence photos ((**C**); 10×), red is JC-1 aggregates and green is JC-1 monomers. ^##^ *p* < 0.01 compared with the control; ** *p* < 0.01 compared with the model; ^$^ *p* < 0.05 compared with A1 group; and ^n.s.^ *p* > 0.05.

In this study, the results show a notable increase in the green fluorescence intensity alongside a reduction in the red fluorescence intensity after UVB damage, as seen in Figure 4. Specifically, the model group exhibited a substantially higher green fluorescence intensity compared with the control group, indicative of a significant decrease in the ΔΨm after UVB irradiation.

The loss of ΔΨm resulted in the excessive accumulation of ROS, which impaired the mitochondrial membranes and led to a decrease in the ΔΨm. In contrast, both the A1 and A2 groups displayed an elevated ΔΨm, signifying a recovery of mitochondrial function post-UVB damage. This resulted in a decrease in damaged cell localization in the mitochondrial membrane and a subsequent reduction in cell apoptosis. 

It has been reported that extracts from edible or medicinal mushrooms can increase the ΔΨm. The polysaccharides in *Hericium erinaceus* mycelium were found to improve mitochondrial function [61]. Similar functions were reported when triterpenoids and meroterpenoids from *Ganoderma resinaceum* were studied [62]. Meanwhile, some wild mushrooms such as *Chlorophyllum molybdites* and *Agaricus endoxanthus* showed potential gastrointestinal and intestinal toxicities, partly because of an inhibited ΔΨm [63].

### 2.5. Effects of A1 and A2 on the Expression Levels of Inflammatory Factors

**Figure 5:**
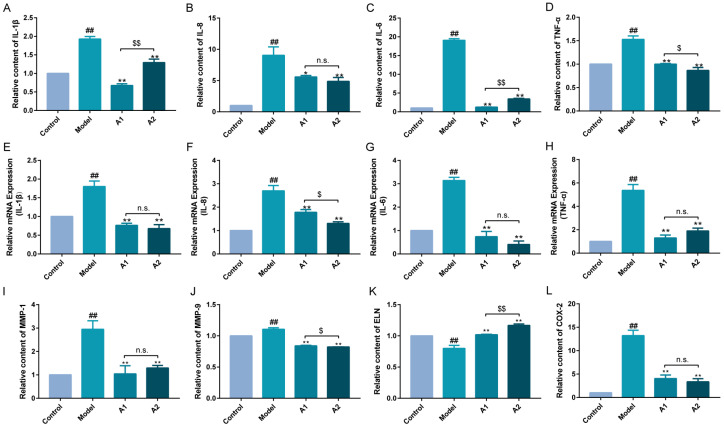
The relative protein and gene expression changes after the repair of A1 and A2 upon UVB injury. The relative contents of MMP-1 (**I**), MMP-9 (**J**), ELN (**K**), and COX-2 (**L**) in HaCaT cells induced by UVB irradiation. Proteins: IL-1β (**A**), IL-8 (**B**), IL-6 (**C**), and TNF-α (**D**). Genes: IL-1β (**E**), IL-8 (**F**), IL-6 (**G**), and TNF-α (**H**). ^##^ *p* < 0.01 compared with the control; * *p* < 0.05 and ** *p* < 0.01 compared with the model; ^$^ *p* < 0.05 and ^$$^ *p* < 0.01 compared with A1 group; and ^n.s.^ *p* > 0.05.

A heightened secretion of pro-inflammatory factors, encompassing interleukins and tumor necrosis factor, signals the initiation of cellular inflammatory responses. As delineated in Figure 5, UVB irradiation led to a marked increase in the protein secretion and gene transcription of IL-1β, IL-6, IL-8, and TNF-α in the model group. In contrast, both the A1 and A2 groups displayed a substantial reduction in the levels of these inflammatory factors.

This anti-inflammatory property of ABP may also be of importance for the mushroom’s medicinal effects on tumors, allergies, and Parkinson’s disease in vivo and in vitro [64,65,66], all of which are inflammatory conditions. These findings once again highlight the therapeutic potential of ABP.

These findings underscore the ability of ABP to effectively inhibit the secretion and expression of inflammatory factors, thus achieving an anti-inflammatory effect.

### 2.6. Influences of A1 and A2 on MMP-1, MMP-9, ELN, and COX-2

MMPs are also mediators of the vicious cycle of inflammation, responsible for the degradation of various components in the extracellular matrix (ECM). The broad role of MMPs may affect not only ECM remodeling but also molecular signaling involved in inflammation and repair, which complicates the interpretation of these findings [67].

As shown in Figure 5, the results show that the contents of MMP-1, MMP-9, ELN, and COX-2 were significantly increased after UVB damage and were significantly decreased after A1 and A2 treatment for 24 h. After UVB stimulation, the levels of MMPs were found to be elevated, and this finding was also documented in our investigation on the fermentation broth of *Laminaria japonica* [43]. The inhibitory effect of A2 on MMP-9 was more significant than that of A1. The promoting effect of A1 on ELN was more significant than that of A2, and the inhibitory effects of A1 and A2 on MMP-1 and COX-2 were not notably distinct.

### 2.7. Expression of JAK-STAT Pathway-Related Genes

The mammalian JAK-STAT signaling pathway comprises four Janus kinase domain-containing proteins and signal transducers and activators of transcription (STATs) [68]. The JAK-STAT signaling pathway plays a critical role in transducing signals from various cytokines, such as interleukins and interferons, to achieve distinct transcriptional outcomes [69].

**Figure 6:**
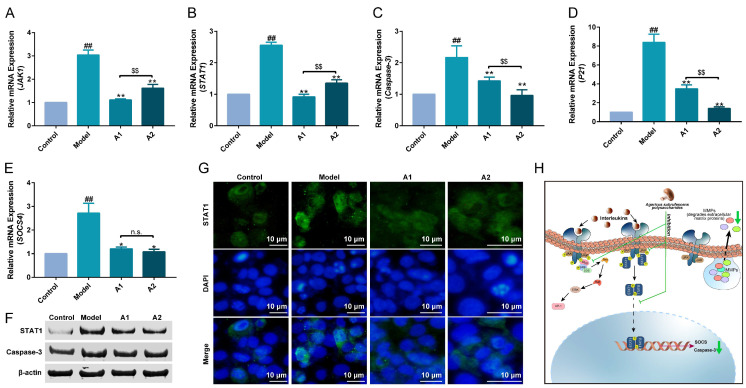
The relative expressions of JAK-STAT signaling pathway-related genes and the levels of STAT1 and caspase-3. JAK1 (**A**), STAT1 (**B**), caspase-3 (**C**), P21 (**D**), and SOCS1 (**E**). The HaCaT cells were subjected to UVB irradiation and treated with A1 and A2, followed by the quantification of STAT1 and caspase-3 protein levels using immunoblotting (**F**) and immunofluorescence (**G**), green is STAT1 and blue is DAPI. The mechanism diagram of anti-photoaging effect of ABM (**H**). ^##^ *p* < 0.01 compared with the control; * *p* < 0.05 and ** *p* < 0.01 compared with the model; ^$$^ *p* < 0.01 compared with A1 group; and ^n.s.^ *p* > 0.05.

In this study, the related gene expressions of the JAK-STAT signaling pathway were detected by qPCR. As shown in Figure 6, the purified polysaccharides A1 and A2 of ABM could inhibit the accelerated expressions of the genes JAK1 and STAT1 under UVB irradiation. Furthermore, the effect of A1 was more significant than that of A2, probably because of the rich monosaccharide composition of A1, which may activate more targets of action. The downstream genes caspase-3, P21, and SOCS1 (suppressor of cytokine signaling 1) were also detected. UVB irradiation induced cellular apoptosis and up-regulated the expression of the pro-apoptotic gene caspase-3, whereas the fermentation broth of *Laminaria japonica* exhibited anti-apoptotic effects by down-regulating the expression of this gene [43]. It was also found that *Entada phaseoloides* effectively mitigated UVB-induced oxidative damage and cell apoptosis through the regulation of caspase-3 and COX-2 gene expression [70]. The P21 gene is a cyclin-dependent kinase inhibitor located downstream of the P53 gene, which is closely related to the cell cycle and cell senescence. SOCS is a family of proteins that inhibit cytokine responses and JAK-STAT pathway activation in a feedback loop. In this study, UVB irradiation accelerated the expressions of caspase-3, P21, and SOCS1. The purified polysaccharides A1 and A2 showed excellent inhibition of these three discussed gene expressions. Moreover, A2 showed relatively greater repression of P21 and caspase-3 than A1.

The protein expression levels of STAT1 and caspase-3 were significantly up-regulated in the UVB damage model, as depicted in Figure 6F. A1 and A2 exhibited inhibitory effects on them (Figure 6F). The immunofluorescence results of STAT1 are presented in Figure 6G. The STAT1 antibody exhibited green fluorescence, while DAPI staining generated a blue fluorescence signal for nucleus visualization. There was a significant increase in the green fluorescence intensity observed in UVB-damaged HaCaT cells, indicating a substantial up-regulation of STAT1 protein expression compared with the control group. Notably, both the A1 and A2 groups displayed significantly lower levels of green fluorescence intensity than that observed in UVB-damaged HaCaT cells (Figure 6G).

Based on the results obtained above, Figure 6H represents the mechanism diagram of ABP’s anti-photoaging effect, which is further discussed in the Discussion.

## 3. Discussion

Ultraviolet (UV) radiation is classified into UVA (315–400 nm), UVB (280–315 nm), and UVC (100–280 nm) radiation [71]. UVB radiation is 1000 times more carcinogenic than UVA radiation and contributes to skin aging. It penetrates the epidermal layer, giving rise to ROS, which inflict severe oxidative damage on skin proteins, lipids, and DNA, thus accelerating aging processes [25]. In addition, skin inflammation occurs, accompanied by the damage of the skin barrier [43]. During the inflammatory phase, cellular biomarkers are activated to block pathogen invasion and reduce damage to the site of inflammation [72]. The responses to inflammation are some of the main mechanisms for clearing away external or internal stimuli [9,15,25,50].

ABM, a Brazilian fungus with extensive medicinal and nutritional utilization [73,74], is recognized for its robust in vitro ability to scavenge free radicals and superoxide anions and holds great promise for mitigating these detrimental effects. The existing studies have primarily focused on its anti-tumor and immunomodulatory attributes [75]. Studies related to photodamage remain relatively underexplored. The investigation of plant polysaccharide extraction and activity remains a crucial area of study [76].

ABPs are ABM’s main biological components, which provide support for the treatment or prevention of tumors, diabetes, and inflammatory diseases [15,36,75,77,78,79,80]. There are similarities between the structures of ABPs. In the present study, the main monosaccharide component of A1 and A2 was glucose, which was similar to the polysaccharides obtained from the fruitbody [36]. The infrared spectroscopy showed C-H, C-O, and O-H bonds in this study. Additional carboxyl (COOH) and carbonyl (C=O) absorption peaks were also detected, which was consistent with the literature [77]. Apart from glucose, mannose and galactose were the other two kinds of monosaccharides that existed in large numbers in the ABP structures [77]. Molecular weight is a key parameter for the activity of polysaccharides. The relationships between the molecular weights of polysaccharides and their functions still need to be analyzed in detail. Another key factor, the type of glycosidic bond, was not studied in this study. This was the limitation of our research. Further studies will be performed in the future.

The mechanisms of action of ABM extracts or polysaccharides are mainly related to the reduction in inflammatory responses [15,80,81,82]. In this study, ABP was found to play a vital role in triggering anti-inflammatory responses to cellular damage. Both A1 and A2 significantly reduced the production of intracellular inflammatory factors (IL-1β and TNF-α) induced by UVB, concurrently enhancing skin barrier function. Their potent anti-inflammatory attributes were consistent with the findings reported in [83,84]. MMPs, key contributors to inflammation, play a pivotal role in ECM degradation [85]. After UVB irradiation, MMP activity escalated in skin cells, suggesting potential photoprotection via MMP inhibition. A1 and A2 significantly inhibited MMP-1 and MMP-9 levels in UVB-damaged cells while increasing the ELN content, underscoring ABP’s anti-inflammatory effects.

Antioxidant and anti-inflammatory activity and mitochondrial health are always interrelated in overcoming oxidative stress-induced damage, hence becoming important targets of anti-aging, anti-UV, and anti-photodamage therapy [61,85,86,87,88]. Mitochondrial function, a critical indicator of cell status, is reflected in the mitochondrial membrane potential [59]. UVB-induced damage reduced cell viability and the mitochondrial membrane potential and promoted cell apoptosis in [89]. Some active compounds showed excellent functional effects by moderating mitochondrial function. Resveratrol induced mitochondrial activity and biogenesis, thus increasing ATP production, in [90]. The antioxidant Trolox was reported to have roles in the mitochondrial membrane potential and ATP production [91]. In this study, ABP exhibited a remarkable ability to enhance the ΔΨm in damaged cells, thus effectively mitigating the occurrence of cell apoptosis.

Moreover, wound healing and injury repair promote the regression of inflammation by restoring barrier function [92]. Wound healing assays demonstrated ABP’s ability to improve cell migration and restore UVB-induced HaCaT cells, suggesting its efficacy against UVB-induced skin photodamage and cell dysfunction, including oxidative damage, inflammation, skin barrier function, and mitochondrial function. The results underscore its potential for skin care applications against photodamage.

The JAK-STAT signaling pathway consists of the JAK and STAT gene families and is a common signal transduction pathway activated by many cytokines [93]. It is responsible for immune functions and the homeostasis of the organism. The activated JAK-STAT signaling pathway up-regulates the expression of ILs, such as IL-6, IL-17, IL-22, and INF-c, thus causing skin diseases, such as atopic dermatitis and psoriasis [69,94]. Some reported ingredients play health functions in a JAK-STAT signaling-related manner. Luteolin can suppress proinflammatory mediators and regulate various signaling pathways, including the JAK-STAT and TLR signaling pathways [95]. *Auricularia auricula* extracts regulated intestinal lipid metabolism and liver thermogenesis by regulating the expression of JAK-STAT signaling-related genes in [96]. In this study, UVB irradiation activated the JAK-STAT signaling pathway, thus causing apoptosis and the over-expression of inflammatory factors. A1 and A2 gave rise to a reduction in the expression of genes in the JAK-STAT signaling pathway, which suggested a decreased inflammatory response (Figure 6H).

However, further investigations, including high-throughput transcriptome technologies like RNA-Seq, will shed light on related signaling pathways and gene interactions, offering a more comprehensive understanding of fungal polysaccharides’ mechanisms of protecting against UVB damage. This effort aims to unravel the anti-photodamage pathway of ABM, advancing its application in food, health products, and skincare.

## 4. Materials and Methods

### 4.1. Materials

HaCaT cells were sourced from the Cell Resource Centre at Beijing Union Medical College (Beijing, China). Dulbecco’s modified Eagle’s medium (DMEM), fetal bovine serum (FBS), phosphate-buffered saline (PBS), penicillin, streptomycin, and 0.25% trypsin (containing EDTA) were procured from Gibco Life Technologies (Carlsbad, CA, USA). Dexamethasone was acquired from Sinopharm Chemical Reagent Co., Ltd. (Beijing, China). PMSF buffer for total protein extraction, the First Strand cDNA Synthesis Kit, the Fast Super EvaGreen^®^qPCR Master Mix, and CCK-8 kits were obtained from Biorigin (Beijing, China) Inc. ELISA kits (IL-1β, IL-8, IL-6, TNF-α, AQP3, KLK-7, FLG, MMP-1, MMP-9, ELN, and COX-2) were all purchased from Wuhan Cloud Clone Technology Co., Ltd. (Wuhan, China). The mitochondrial membrane potential assay kit was sourced from Beyotime Biotechnology Co., Ltd. (Beijing, China). STAT1 and caspase-3 were acquired from Abcam (Beijing, China) Biomedical Technology Co., Ltd.

The UV crosslinker (model SCIENTZ03-II) was obtained from SCIENTZ Biotechnology Co., Ltd. (Ningbo, China).

### 4.2. Extraction, Separation, and Purification of ABP

ABM was cultured at 28 °C and 150 rpm for 5 days to obtain the ABM fermentation broth. ABP was extracted using the ethanol precipitation methods according to the report [25]. Briefly, the fermentation broth was collected after centrifugation and then mixed with a three-fold volume of absolute ethanol, followed by storage overnight at 4 °C. After centrifugation, the supernatant was discarded and diluted with 100 mL of deionized water. Residual protein removal from ABP was performed using the Sevage method [97,98], and the efficacy of the removal was quantitatively evaluated. The resulting sediment underwent alcohol precipitation once again. The precipitate obtained was dissolved in ultrapure water, dialyzed using a membrane with a molecular weight cut-off of 10 kDa for 48 h, and, finally, freeze-dried to yield crude ABP.

DEAE-52 cellulose column chromatography was adopted to isolate and purify the ABP, and then the eluents were collected by step elution with 0, 0.1, 0.3, and 0.5 mol/L NaCl at a flow rate of 1.0 mL/min [99]. The volume of each collection tube was 10 mL.

An elution curve was constructed, plotting the absorbance of each eluted fraction against the concentration of NaCl, while the ordinal numbers of the eluted fractions were represented along the horizontal coordinate axis [100]. Following this, fractions corresponding to the same elution peak were collected together, undergoing subsequent procedures such as concentration, dialysis, and lyophilization. There were two isolated components, and they were identified as A1 and A2. These purified components were subsequently used for detailed experimental investigations, ensuring the reliability and consistency of the research findings.

### 4.3. UV–Vis

The UV–visible spectrum analysis within the range of 200 to 800 nm was conducted for A1 and A2 at a concentration of 1 mg/mL, separately.

### 4.4. FT-IR

The mixture of 1 mg of A1 and A2 was finely ground into a powder, which was then combined with 100 mg of KBr (particle size: 200 mesh) before being compressed into a thin sheet using a mold. Additionally, the molecular structures of A1 and A2 were analyzed with a Fourier-transform infrared spectrometer within the range of 4000 to 400 cm^−1^, with a resolution of 1 cm^−1^ [101,102].

### 4.5. SEM

Moreover, the morphological characteristics of A1 and A2 were examined using an FEI Nova Nano SEM 450 instrument (FEI Company, Hillsboro, OR, USA) in the vacuum mode [43]. A total of 5 mg of each sample was immobilized on the SEM stub, coated with a 10 nm layer of gold, and observed at magnifications ranging from 250 to 1000×, with the accelerating voltage set at 5.0 kV.

### 4.6. AFM

For microstructural investigations, an advanced Dimension Icon-Three-directional closed-loop scanner (Bruker AXs, Saarbrücken, Germany) was employed for atomic force microscopy (AFM) imaging. The sample was dissolved in distilled water to obtain a solution of 10 μg/mL, and the supernatant was filtered through a 0.45 μm membrane filter. Subsequently, we deposited 20 μL of the solution onto freshly prepared mica surfaces and allowed it to air-dry for 24 h at room temperature. For imaging purposes, we employed a gas-phase probe (ScanAsyst-Air, Bruker AFM Probes, Tokyo, Japan) operating in the intelligent imaging mode. The scanning mode utilized was ScanAsyst, with the scanning speed set at 1 Hz over an area of 2.5 μm × 2.5 μm. The scanning range covered an area of 90 µm × 90 µm in the XY-direction, achieving an impressive overall noise level below the 0.03 nm RMS value [25].

### 4.7. GPC and Monosaccharide Composition Analysis

The molecular weight distribution of A1 and A2 was determined using gel permeation chromatography with light scattering and refractive index detectors (GPC-LS-RI) following referenced protocols [25,43]. A high-performance liquid chromatography (HPLC) system equipped with an Xtimate C18 column (4.6 × 200 mm; 5 µm) was employed for the monosaccharide composition analysis.

Mannose, ribose, rhamnose, glucuronic acid, galacturonic acid, n-acetyl-glucosamine, glucose, n-acetyl-galactose, galactose, xylose, arabinose, and fucose (Sigma, Beijing, China; >99%) were selected as the standard control for comparison.

Briefly, samples were hydrolyzed in nitrogen with 2 M trifluoracetic acid (TFA) for 4 h at 120 °C. Following evaporation under vacuum for the removal of TFA, the samples were resuspended in 3 mL of water. An amount of 250 µL of hydrolysate or the mixed control solution was transferred into a 5 mL EP tube. Then, 250 µL of 0.6 M NaOH and 500 µL of 0.4 M 3-methyl-1-phenyl-2-pyrazoline-5-one (PMP)-methanol were added to start the reaction at 70 °C for 1 h. After cooling for 10 min in cold water, 500 µL of 0.3 M HCl was added for neutralization. One milliliter of chloroform was then added to extract the derivatized sample. The mobile phase consisted of an 83% 0.05 M dihydrogen phosphate solution (pH 6.70, adjusted with NaOH) and 17% acetonitrile. The samples were detected using HPLC at a wavelength of 250 nm, a flow rate of 1 mL/min, and a temperature of 30 °C. Table 3 and Figure 2G represent the standard sugar profile information and the calculation formula for determining the concentration of each monosaccharide.

### 4.8. Cell Culture and Toxicity Assay

HaCaT cells were cultivated in DMEM supplemented with 10% FBS and 1% penicillin–streptomycin, maintaining a controlled incubation environment at 37 °C with 5% CO_2_ for optimal growth. Medium renewal was conducted at 48 h intervals to sustain cellular conditions. The passage of cells was initiated upon reaching an 80% confluence level.

Cell viability was measured according to the previous method [21], with adherence to the provided kit instructions. The HaCaT cells were seeded in 96-well plates at a density of 1 × 10^5^ cells per well. The viability assessment encompassed diverse treatment conditions, including varying concentrations of ABP (0–1000 μg/mL) and UVB exposure levels (0–40 mJ/cm^2^ at 310 nm) with a UV cross-linker (UV07-II; Ningbo, China). The CCK8 assay was employed, and optical density (OD450) measurements were recorded utilizing a microplate reader (Thermo Fisher Scientific Instruments Ltd., Beijing, China). The determination of the half-maximal inhibitory concentration (IC50) values was employed to establish the UVB-induced experimental model.

### 4.9. The Secretions of Inflammatory Factors and Related Enzymes Were Determined by ELISA

HaCaT cells were cultured in a 6-well plate at a density of 5.0 × 10^5^/well. After the pre-irradiation with UVB, the cells were post-treated with the sample at a certain concentration for 24 h. The secretions of IL-1β, IL-8, IL-6, TNF-α, AQP3, kallikrein-7 (KLK-7), FLG, matrix metalloproteinase-1 (MMP-1), matrix metalloproteinase-9 (MMP-9), elastin (ELN), and cyclooxygenase-2 (COX-2) were determined according to the manufacturer’s instructions. Dexamethasone (DEX) at a concentration of 1 μM was used as the positive control.

### 4.10. Determination of Cell Migration Capacity

HaCaT cells were cultivated in 6-well plates until reaching 80% confluence. Subsequently, parallel linear scratches were made three times per well, mimicking controlled wounds. These scratch-incubated wells were then subjected to UVB exposure, as well as A1 and A2 stimulation, for 24 h.

The healing progress of these cell scratches was meticulously observed at both the initial 0 h time point and again after 24 h. Measurements were taken to calculate the percentage of the healed area between the cell scratches. The calculation formula was as shown below:

Cell scratch healing rate = 100%× [Cell scratch area at the beginning (0 h) − Cell scratch area after 24 h (24 h)]/Cell scratch area at the beginning (0 h)

### 4.11. Mitochondrial Membrane Potential Measurements

The cell culture procedure remained consistent with the methodology described in Section 4.9. HaCaT cells were cultivated in 6-well plates and then subjected to UVB irradiation to induce cellular damage. Subsequently, the cells were treated with A1 and A2 for 24 h, following the guidelines provided by the kit manufacturer, and observed with a fluorescence microscope, and the fluorescence value was measured with a fluorescent enzyme label [60,103].

The assessment of the mitochondrial membrane potential (ΔΨm) was carried out using a mitochondrial membrane potential detection kit containing JC-1 (Beyotime Biotechnology Co., Ltd., Beijing, China). This kit assesses the ability of JC-1 to selectively enter mitochondria, with its color changing in response to variations in the membrane potential. When the ΔΨm increases, the color of JC-1 shifts from green to red; conversely, a decrease in the ΔΨm results in the reverse color change.

### 4.12. RT-qPCR

After treatment with A1, A2, and UVB irradiation, total cellular RNA was extracted using the TRIzol RNA extraction reagsent. Then, cDNA was synthesized according to the manufacturer’s instructions. RT-qPCR was performed using the Fast Super EvaGreen^®^qPCR Master Mix (QuantStudio3, Thermoscientific, Shanghai, China).

The β-actin gene served as an internal control (the primer sequences are presented in the Supplementary Materials, Table S1). We analyzed the relative gene expression levels using the 2^−ΔΔCT^ method [104].

### 4.13. Immunofluorescence Analysis

The HaCaT cells were fixed using a solution containing 4% paraformaldehyde, followed by treatment with TritonX-100 reagent to enhance the permeability of the cell membranes. BSA was utilized for the sealing process. The primary antibody (STAT1) was incubated overnight at a temperature of 4 °C, while the diluted secondary fluorescent antibody was added in darkness at a dilution ratio of 1:500 and incubated for a duration of 1.5 h. Light exposure during this step was avoided, and subsequently, a solution containing DAPI dye was introduced. An anti-fluorescence quenching agent was then applied before covering the bottom plate and observing the protein fluorescence staining under an inverted fluorescence microscope.

### 4.14. Western Blot

The treated cells were collected and lysed with RIPA lysis buffer. The cell debris was removed at 4 °C by centrifugation at 1000× *g* for 10 min. The protein concentration in the cells was measured using Bradford reagent with bovine serum albumin as a standard. Cell lysates containing equal amounts of total protein were separated using 12% SDS-PAGE gel and then transferred to a PVDF membrane and blocked with 5% skimmed milk/Tris-buffered saline containing Tween 20 (TBST) at room temperature for 1 h. The membranes were incubated with primary antibodies against β-actin, STAT1, and caspase-3 in 5% milk/TBST overnight at 4 °C. The membranes were washed 3 times with TBST for 10 min each. The second antibody was incubated with horseradish peroxidase at room temperature for 1 h. For the analysis, the membranes were incubated with ECL luminous fluid and exposed to X-ray films in a dark room. The pre-stained protein molecular weight marker (10 to 180 kDa) was used for the protein size calculation.

### 4.15. Statistical Analysis

All experiments were performed in triplicate, and the data are presented as means ± standard deviations. Image J version 2.0 (National Institutes of Health, Bethesda, MD, USA) software was used to process the images. The data were analyzed by SPSS 17.0 software and GraphPad Prism 9.0 (GraphPad Software, San Diego, CA, USA) software. One-way analysis of variance was used for the significance of differences among groups (*^, #, $^ *p* < 0.05, **^, ##, $$^ *p* < 0.01, and ^n.s.^ *p* > 0.05).

## 5. Conclusions

There were two highly purified polysaccharides, designated as A1 and A2, obtained after the isolation and purification process. Subsequent comprehensive structural and compositional analyses revealed the predominant presence of glucose, galactose, and arabinose as major constituents in A1 and A2. Notably, both A1 and A2 exhibited robust anti-inflammatory properties in decreasing pro-inflammatory factors, simultaneously mitigating mitochondrial dysfunction, and enhancing the cell migration capacity. Furthermore, these polysaccharides exhibited significant reductions in MMPs and an obvious increase in ELN levels. At the gene level, A1 and A2 can inhibit the activation of the JAK-STAT signaling pathway by down-regulating the expression of JAK1, STAT1, caspase-3, P21, and SOCS1 genes, thus playing a role in anti-inflammatory processes and barrier repair.

## Figures and Tables

**Table 1:** Molecular weight determinations of A1 and A2.

	A1	A2
Peak limits (min)	21.921–24.953	25.237–26.727
Mw	1.5 × 10^4^	6.5 × 10^4^
Mz	2.4 × 10^4^	9.1 × 10^4^
Mw/Mn	1.282	1.297
Mz/Mn	2.011	1.818

**Table 2:** Monosaccharide components of A1 and A2.

Monosaccharide Ratio (%)	A1	A2
Mannose	3.918	0.640
Ribose	0.976	0.191
Rhamnose	3.077	0.716
Glucuronic acid	1.353	0.285
Galacturonic acid	0.617	0.571
Glucose	61.478	78.373
Galactose	21.460	11.108
Xylose	——	0.977
Arabinose	5.832	6.925
Fucose	1.290	0.215
Total	100.000	100.000

Note: *N*-acetyl-glucosamine and *N*-acetyl-galactose were not detected in both of the fractions.

**Table 3:** The standard sugar profiles (250 nm).

**Peak No.**	**RT**	**Standard Sugar**	**Molecular Weight**	Standard Curve *
1	15.949	Mannose	180.16	y = 29,549.99x
2	20.586	Ribose	150.13	y = 35,195.08x
3	21.669	Rhamnose	164.16	y = 24,606.19x
4	26.147	Glucuronic acid	194.14	y = 23,994.21x
5	30.011	Galacturonic acid	194.14	y = 25,574.43x
6	31.584	*N*-acetyl-glucosamine	221.21	y = 21,259.33x
7	34.458	Glucose	180.16	y = 28,273.87x
8	36.462	*N*-acetyl-galactose	221.21	y = 27,966.82x
9	39.481	Galactose	180.16	y = 33,401.43x
10	41.267	Xylose	150.13	y = 37,008.62x
11	42.925	Arabinose	150.13	y = 40,595.71x
12	48.109	Fucose	164.16	y = 34,153.37x

Note: * x represents the concentration of each monosaccharide (μg/mL), and y represents the peak area of each monosaccharide.

## Data Availability

The data used and/or investigated during the present study are available from the corresponding author upon reasonable request.

## Supplementary Materials

The following supporting information can be downloaded at https://www.mdpi.com/article/10.3390/ijms25094676/s1.
