# Supplementary material for: Anti-Photodamage Effect of Agaricus blazei Murill Polysaccharide on UVB-Damaged HaCaT Cells
Source: Int J Mol Sci. 2024 Apr 25;25(9):4676. doi: 10.3390/ijms25094676 (PMC11083510; doi:10.3390/ijms25094676)
Supplement: Supplementary file 1 [file ijms-25-04676-s001.zip › ijms-2960788-supplementary.pdf]

Table S1. Primer sequences for Real-Time PCR

Gene	Direction	Primer pair sequence (5'→3')
IL-1β	F	CCTGAGCTCGCCAGTGAAA
	R	GTGGTGGTCGGAGATTCGTA
IL-8	F	AAGATGTGAAGCTGACGCAGA
	R	AGAATTGAGCTGAGCCTTGG
IL-6	F	GATGAGTACAAAAGTCCTGATCCA
	R	CTGCAGCCACTGGTTCTGT
TNF-α	F	CACAGTGAAGTGCTGGCAAC
	R	AGGAAGGCCTAAGGTCCACT
JAK1	F	TCTGCATCGAGCGCACAAA
	R	GTAGGGTTGAGGGACATCTGC
STAT1	F	GTGCACAGAATCCTCCACCA
	R	ACTGTGCCAGGTACTGTCTG
Caspase-3	F	AGGACTCTAGACGGCATCCA
	R	CAGTGAGACTTGGTGCAGTGA
P21	F	TGCCGAAGTCAGTTCCTTGT
	R	AGATTCTGGCTGACTGCTCG
SOCS1	F	ACATGGTCCTCTGCGTTCA
	R	GGAGCGCAGCAGTCTAAAAC
β-actin	F	TGGCACCCAGCACAATGAA
	R	CTAAGTCATAGTCCGCCTAGAAGC
